# Reduced Context Effects on Retrieval in First-Episode Schizophrenia

**DOI:** 10.1371/journal.pone.0010356

**Published:** 2010-04-27

**Authors:** Lucia M. Talamini, Lieuwe de Haan, Dorien H. Nieman, Don H. Linszen, Martijn Meeter

**Affiliations:** 1 Department of Psychology, University of Amsterdam, Amsterdam, The Netherlands; 2 Academic Medical Center, Department of Psychiatry, University of Amsterdam, Amsterdam, The Netherlands; 3 Department of Cognitive Psychology, VU University, Amsterdam, The Netherlands; Chiba University Center for Forensic Mental Health, Japan

## Abstract

**Background:**

A recent modeling study by the authors predicted that contextual information is poorly integrated into episodic representations in schizophrenia, and that this is a main cause of the retrieval deficits seen in schizophrenia.

**Methodology/Principal Findings:**

We have tested this prediction in patients with first-episode schizophrenia and matched controls. The benefit from contextual cues in retrieval was strongly reduced in patients. On the other hand, retrieval based on item cues was spared.

**Conclusions/Significance:**

These results suggest that reduced integration of context information into episodic representations is a core deficit in schizophrenia and one of the main causes of episodic memory impairment.

## Introduction

Memory is regarded as one of the major areas of cognitive deficit in schizophrenia. Particularly pronounced impairments are observed in episodic memory [1]–[3]. They include moderate to severe deficits in free recall, lesser ones in cued recall and a small, but significant, deficit in recognition [1], [4]. These impairments are not due to either faster forgetting [1], [2], [4]–[6] or increased sensitivity to interference [7]; they are relatively unresponsive to medication [1], [8]–[10], not substantially modulated by age, severity of psychopathology or duration of illness [1], and can be identified in approximately 75% of patients [11].

There is now substantial evidence that episodic memory deficits in schizophrenia are largely due to abnormal encoding, even though retrieval may not be entirely spared [1], [2], [4]–[6], [12], [13]. Indeed, several theories have proposed a binding deficit in schizophrenia, whereby event components are poorly linked during encoding. As a consequence, patients would be especially impaired on tasks that rely strongly on such links [14]–[16].

One such account has focused on deficient binding between two kinds of information in memory: object information and spatial contextual information. These two types of information are processed in different brain areas and reach the hippocampus over largely separate routes [17], [18]. Talamini et al. [15], [16], [19] showed, using a computational model, that reduced connectivity observed in the medial temporal lobe of patients with schizophrenia [20] leads to poor integration of these two event components. Importantly, this is related to an overrepresentation of object information at the expense of spatial contextual information. The model was shown to mimic both the memory deficits and the contextual processing deficits associated to schizophrenia [15], [16].

A specific prediction of the model holds that the normal effects of context on retrieval should be strongly reduced in patients with schizophrenia, as this type of information is poorly integrated into the episodic representation at the time of encoding. As a consequence, recall of objects based on intra-object cues (e.g. a word stem or category cue) should be relatively spared, while recall based on context cues (e.g. the environment where the object was encountered) should be severely impaired. According to this viewpoint, free recall is relatively impaired in schizophrenia, because it requires one to reinstate the learning context and use it to retrieve item information. Recognition, on the other hand, relies to a large extent on memory for individual items [21]–[23] and is therefore less impaired.

We here test the aforementioned prediction using a new paradigm, in which each item is studied against a background picture that functions as its unique context. At test, half of the items are presented in the same context, while half of the object-context pairs are rearranged to produce new combinations. This creates two conditions: one in which unique contextual information is available to aid object retrieval, and one in which it is not. In comparing context effects on retrieval we are using one task, namely recall of words from word stem cues. This is in contrast to other studies of contextual binding [14], [24], where comparison is across tasks that may have differed in difficulty and retrieval demands.

As predicted by our model, we expect that the context manipulation will have a much smaller effect in patients with schizophrenia than in healthy participants. Moreover, we expect that patients will show a preferential performance impairment when context aids retrieval. What makes this prediction counterintuitive is that patient's deficits should thus be larger when recall is relatively easy (with matching context) than when recall is difficult (with nonmatching context), whereas a standard finding in neuropsychology is that patient's deficits are larger in more difficult tasks. Finally, we predict that recall deficits related to deficient context processing should far outweigh any overall recall deficits on the task.

A second aim of this study was to assess episodic binding at the beginning of the illness. All studies on this function thus far concerned chronically ill patients [14], [24]. Here we assess the effects of contextual information on retrieval in patients that recently suffered a first psychotic episode and were diagnosed with schizophrenia, and in healthy controls, matched on sociodemographic variables and estimated IQ. Thus, potential effects of long-term hospitalizations, long-term medication [25], [26], or progressive structural brain abnormalities [27]–[29] on task performance should be minimal.

## Methods

### Ethics statement

This study was conducted according to the principles expressed in the Declaration of Helsinki. The study was approved by the Ethics Committee of the University of Amsterdam. All subjects gave written informed consent.

### Participants

Nineteen patients (4 in-patients and 16 out-patients), which had recently experienced a first psychotic episode were recruited at the Early Psychosis Unit of the Academic Medical Centre of Amsterdam. Inclusion criteria for this study were: patients should be able and willing to give written informed consent, have a diagnosis of recent-onset schizophrenia or a related disorder according to DSM-IV (APA), be between 16 and 26 years of age and be able to understand and speak Dutch. Exclusion criteria were: diagnosis of a primary alcohol- or drug-related psychosis, a demonstrable brain, neurological or endocrine disease, mental retardation and any current or recent morbidity with psychiatric or neurological diagnoses other than schizophrenia. Additional exclusion criteria for the healthy subjects were occurrence of schizophrenia, or other schizophrenia spectrum disorders, in first-degree relatives. All subjects had normal, or corrected to normal vision and hearing, and used no recreational drugs during testing and in the 48 hours prior to testing.

Clinical discharge diagnoses according to DSM-IV were made with the use of all available diagnostic information (systematic interviews of patients and parents and previous medical records) by two clinical psychiatrists and two residents, after which the diagnoses were reviewed by a research psychologist and a research psychiatrist (LEAD, [30]). Six patients received a DSM-IV diagnosis of schizoaffective disorder, 13 patients were diagnosed with schizophrenia. All patients were stabilized on antipsychotic medication. Mean dose in chlorpromazine equivalents was 233.2 (SD 130.1). Four patients received an SSRI and 2 patients a benzodiazepine additionally.

Nineteen healthy subjects, carefully matched to control subjects with respect to IQ and sociodemographic factors, were recruited through local announcements and were screened to rule out any current or recent psychiatric history.

Table 1 shows sociodemographic variables and estimated IQ scores for patients and control subjects. IQ was assessed using a short version of the Wechsler adult intelligence scale, third edition (WAIS-III; Dutch translation; [31]). Performance IQ was tested using the symbol substitution and block design subtests, and verbal IQ using the arithmetic/calculus and information subtests. There were no statistically significant differences between groups on any of the reported variables (statistical values are given in the table).

**Table 1:** Demographic characteristics.

	Patients (n = 19)		Controls (n = 19)		Statistics		
	M	SD	M	SD	t	df	Sig.
Age	22.26	3.28	22.68	3.22	0.40	1,36	0.69
IQ	99.11	9.89	101.47	8.6	0.79	1,36	0.44
Gender	3 f/16 m		3 f/16 m				

M = mean, SD = standard deviation, f = female, m = male.

### Paradigm

**Figure 1:**
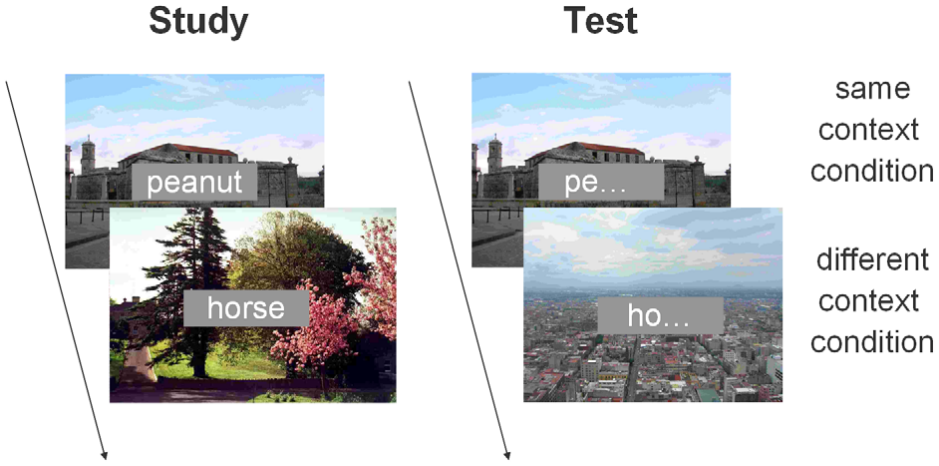
Paradigm used. Participants studied forty words with, as background, a color photograph of an indoor or outdoor scene (picture not to scale). The first test consisted of a cued recall test in which participants had to complete word stems of studied words with a word from the studied list. Half of the word stems were presented with the same scene on the background as during learning (*same context* condition), half with a different scene on the background (*different context* condition). A second test (not shown) was a word recognition test with, again, same or different scenes in the background.

Participants studied 40 concrete nouns of between 5 and 10 letters. Each word was presented on a small gray rectangle (6.5 * 2.1 visual degrees) at the centre of the screen against the background of a color photograph representing a natural or city landscape (see Figure 1). The background scenes contained no distinguishing objects and each list word was presented against a different landscape. Participants were instructed to learn the words on which they would later be tested; learning of the pictures was incidental. The background scenes are thus contextual in the sense that they are not central to the task; moreover, their distinctiveness relies mostly on spatial configural information. Picture-word combinations were randomized anew for each participant. Words were presented twice, in the same order, for 4 seconds with, in between each word, a gray screen with fixation cross presented for 1 second.

Immediately after the last presentation, participants received instructions for the cued recall test. In the test, they were presented with the first two letters of each list word and instructed to finish it by typing the rest of the word from the study list. Order of the word stems was randomized with the proviso that stems for words on the first half of the studied list were also presented in the first half of the test. The word stem cue was presented on the same gray square at centre screen as at study, with again a scene in the background. Half the cues were now combined with the same landscape as at study (*same context* condition), while the other half of the word cue-landscape pairs were rearranged to form new pairs (*different context* condition). The test was self-paced; participants were instructed to respond with an X if they could not remember the word.

Following the cued recall test an old/new recognition test was administered. For the recognition task, the 40 previously learned words were intermixed with 40 foil words (concrete nouns not presented during learning). The attribution of words to the foils and list items was randomized anew for each participant. Again, half of the studied words were presented against the same background as at study (these were the same words as in the recall test), while the other half of the word-landscape pairs was rearranged to form new pairs (these were different combinations than in the recall test). The foil items were also presented against backgrounds viewed during the learning session; each background scene featured behind one foil item. The test was again self-paced; participants responded by pressing the X (‘old’) or N key (‘new’).

### Statistical analysis

Statistical analysis was performed using the SPSS statistical software package (SPSS Inc, Chicago, Illinois). The cued recall and recognition data were analyzed separately, using ANOVA procedures with a between subject factor ‘group’ (healthy; schizophrenia) and within subject factor ‘context condition’ (same context; different context). Post hoc tests were independent samples, two-tailed T-tests. In all tests statistical significance was considered at P<0.5.

In the recognition test one patient responded with ‘new’ to all items. The recognition data of this patient was excluded from statistical analysis.

## Results

**Figure 2:**
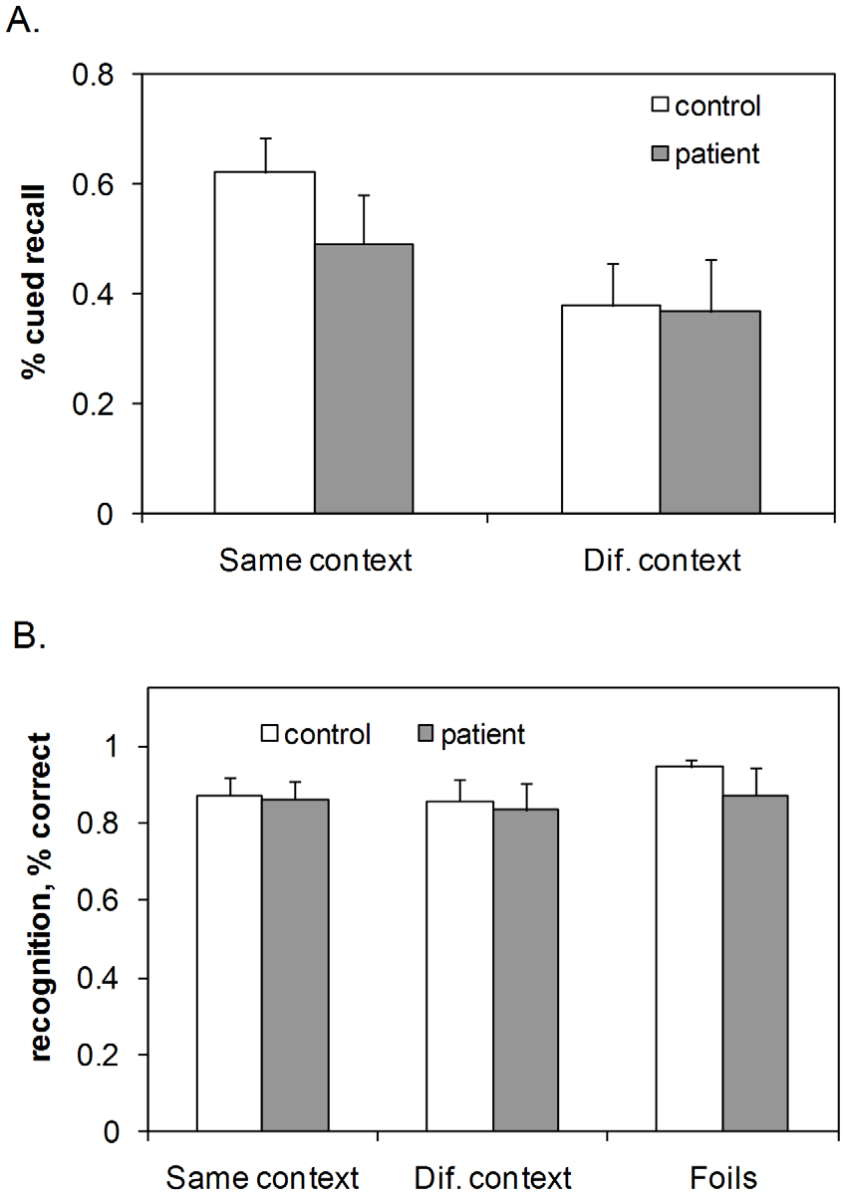
Retrieval performance in the ‘same’ and ‘different’ context conditions. Mean cued recall (a) and recognition (b) of words in the *same context* and *different context* conditions, for patients and matched controls. Error bars give 95% confidence intervals for the means.

Figure 2a shows mean cued recall performance of the two groups of participants for the *same context* and *different context* conditions. An ANOVA on the cued recall data, with a between subject factor ‘group’ (healthy; schizophrenia) and within subject factor ‘context condition’ (same context; different context) showed that memory was better in the same context condition than in the different context condition (main effect of context condition: F(1,36) = 42.05, P<0.0001). There was no main effect of group (F(1,36) = 1.75, P = 0.19), suggesting no substantial overall memory deficit in the patient group. Importantly, there was an interaction between group and context condition (F(1,36) = 4.41, P = 0.043): in healthy subjects word retrieval was aided much more strongly by the presence of the correct background (a 24% benefit over the different context condition) than in the patients (12%). Post-hoc tests showed a substantial difference between groups in the same context condition (t(36) = 2.31, P = 0.027), but none in the different context condition (t(36)<1).

The same analysis was repeated with global IQ score, age and gender as covariates. Of the covariates, only global IQ score interacted significantly with context condition (F(1,33) = 4.67, P = 0.038), reflecting increased use of context information with higher global IQ score. However, this did not alter the outcome of the ANOVA with respect to either the main effects (main effect of context condition: F(1,33) = 7.10, P = 0.012; main effect of group: F(1,33) = 1.14, P = 0.29) or the interaction between group and context condition (F(1,33) = 6.11, P = 0.019).

Figure 2b shows mean recognition performance of the two groups of participants, in terms of hit rates and false alarm rates. As a measure of overall recognition performance we also calculated *d'* (Table 2). In line with expectations, the recognition data showed no effect of the context manipulation. Moreover, there were no significant differences between groups in recognition performance: ANOVA with a between subject factor ‘group’ and within subject factor ‘context condition’ revealed no main or interaction effects on discrimination measure *d'* (main effect of context condition: F(1,35) = 0.37, P = 0.55; main effect of group: F(1,35) = 1.97, P = 0.17; interaction effect: (F(1,35) = 0.008, P = 0.93) or on hit rates (main effect of context condition: F(1,35) = 1.4, P = 0.25; main effect of group: F(1,35) = 1.5, P = 0.23; interaction effect: (F(1,35) = 0.28, P = 0.60). Adding global IQ score, age and gender as covariates to these analyses did not substantially alter these results (all effects n.s.). Consistent with earlier findings in patients [32], [33] and with predictions of our model [16], however, there was a trend towards an increased false alarm rate in patients (t(35) = 1.94, P = 0.06; Figure 2b).

**Table 2:** D' values for the ‘Same picture’ and ‘Different picture’ conditions.

	Patients (n = 18)		Controls (n = 19)	
	M	SD	M	SD
Same picture	2.52	0.95	2.90	0.60
Different picture	2.47	1.14	2.86	0.69

M = mean, SD = standard deviation.

## Discussion

We evaluated context effects on retrieval in a group of patients with first-episode schizophrenia and a group of healthy control participants. The normal benefit from context cues was strongly diminished in the schizophrenic group. Moreover, this impairment far outweighed any overall memory deficit, since performance on overall word recall and on recognition did not differ significantly between patients and controls.

Given the very close match between patients and controls on IQ and sociodemographic variables, confounds in our findings from these variables are unlikely. It is equally unlikely that the contextual processing deficit is secondary to a general memory deficit in the patient group, as no such deficit was found. Interpretations of findings in terms of task difficulty or retrieval effort are also implausible, since patients were impaired only on the easier task condition with the matching context. Finally, there are no floor, ceiling or scaling effects in the current set up. Therefore, our findings show a substantial and selective deficit in contextual memory processing in first-episode schizophrenia.

The absence of a significant recognition deficit in our group of patients (in terms of d' values) is in line with meta-analyses showing relatively spared recognition relative to recall in schizophrenia [1], [34] and with studies showing milder deficits in first-episode patients than in chronically ill samples [13]. However, a few studies in patients with first-episode schizophrenia, using larger samples than our own, did find recognition deficits with respect to healthy patients [35]–[37]. Since mean d' values are slightly lower in our patient sample than in the healthy controls, it might be that a minor recognition impairment was missed due to insufficient power in our study.

Our findings confirm the predictions of the Talamini et al model [15], [16], [19]. According to this model, contextual processing deficits are due to a substantial reduction of connectivity in the mediotemporal lobe in schizophrenia. Such a reduction has been demonstrated by several studies showing massive loss in the density of synaptic and dendritic molecules in the (para)hippocampal region [20], [38], [39], which is in fact also the brain region showing the largest volumetric reduction in schizophrenia. The crucial role of these areas in binding components of events into episodic representations has long been established, and several studies have linked memory deficits in schizophrenia to abnormalities in these regions [40]–[42].

**Figure 3:**
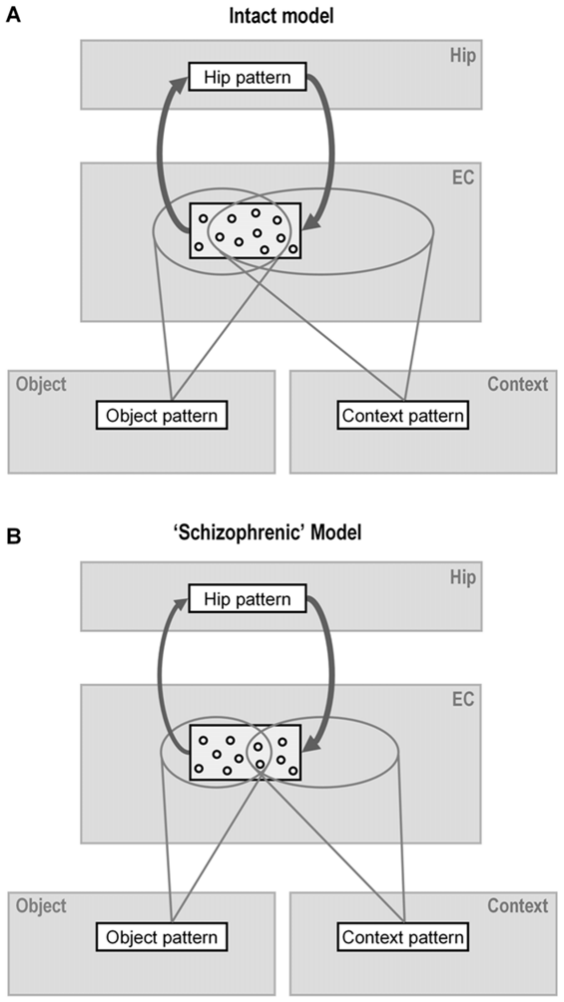
Integration of object and spatial information in the parahippocampal regions of the model. The Talamini et al. model [15], [16], [19] captures the basic organization of the hippocampus and parahippocampal areas in a simplified manner. It consists of four interconnected modules (shown in light grey), representing the hippocampus (Hip), entorhinal cortex (EC), perirhinal cortex (Object) and parahippocampal cortex (Context). Each module consists of many simulated neurons. A presentation of an object and its context activates neural patterns (shown as white rectangles), in all four modules. Only the simulated neurons making up the active pattern in the entorhinal module are depicted (small black circles). (a) In the normal model there is considerable convergence of input connections on entorhinal neurons (overlap area of projections from the active object and context patterns). Thus, when an object-context pairing is being learned, many entorhinal neurons get input from both the object pattern and the context pattern. (b) However, in the ‘schizophrenic model’ the connections between the input layers (Object and Context) and the EC, as well as the connections between the EC and the Hip, are reduced by 50%, in line with studies suggesting substantial hypoconnectivity in these projections [20], [38], [39]. The reduction of the input projections reduces the probability that a given entorhinal neuron receives input from both sources. This favors the inclusion of neurons receiving only context- or only object input in entorhinal representations. Since single object projections are stronger than single context projections (an architecture motivated by both functional and anatomical considerations; see Talamini et al. 2009 [16] and Suzuki et al. 1994 [61]), neurons receiving only object input have a higher chance of winning the competition for activation than neurons receiving only context input. Thus object information gets overrepresented in the entorhinal pattern, at the expense of context information. Due to this circumstance, object cues activate large parts of entorhinal patterns and can lead to retrieval irrespective of context cues. Conversely, isolated context cues activate only a small portion of associated entorhinal patterns, which is often insufficient for successful retrieval.

In our model, reduced mediotemporal lobe connectivity leads to fragmented episodic representations, in which objects are overrepresented at the expense of spatial contextual information. Retrieval is, therefore, much more dependent on object than context cues (Figure 3). What happens during retrieval in schizophrenia, in the condition with the correct background image, is that the background image activates an abnormally small part of a previously learned episodic representation, which is moreover not well connected to the rest of the episodic pattern. Therefore, the contextual background cue contributes little to reactivation of the previously stored representation. On the other hand, the word stem cue activates a larger than normal part of the previously stored representation, thus serving as an efficient retrieval cue in both context conditions.

The aforementioned mechanism also leads to increased false alarms in our model. Indeed, large (or full) cues for familiar objects may activate a previously stored episodic representation sufficiently to lead to recognition, even if the current context cue does not match the representation [16]. In line with our model, false alarms were somewhat increased in our patient sample, although the finding did not reach statistical significance (P = 0.06). Similar findings have been reported by others [32], [33], [43], [44] and have been related to a decreased conscious (or source specific [32], [33]) recollection and a consequent reliance on familiarity in schizophrenia [43]. Our model shows how a specific pattern of neuropathology could, indeed, lead to these deficits.

Previous studies of cued recall in patients with schizophrenia have typically reported sizeable deficits in cued recall [1], which would seem inconsistent with the absence of an overall cued recall deficit in our sample. This may be due to the fact that previous studies have not manipulated context, and are therefore comparable with the ‘same context’ condition, for which we did find deficits in the patient group. Another factor that differentiates our study from others is the use of an intra-object cue; that is, the word-stem cue. As explained in the introduction, such cues should be relatively effective at eliciting retrieval in patients with schizophrenia. We are not aware of other studies assessing word stem-based recall in schizophrenia. In more typical cued recall paradigms the explicit retrieval cue tends to be an extra-object cue (e.g. a paired associate). According to our model, performance on such paradigms would be more dependent on efficient binding of event components than with an intra-object cue. In line with this notion, performance of patients with schizophrenia on typical cued recall tasks tends to be more disrupted than on our currently used paradigm.

The existence of a binding deficit in schizophrenia is consistent with several studies that explicitly investigated memory for new associations between objects, spatial and temporal aspects of an event. Some such studies show severe deficits in tasks in which performance relies entirely on newly formed associative links between stimuli; for instance, in associative recognition, in which item pairs are pitted against recombined pairs [24], [45]. Other studies report schizophrenia-related impairments for retrieval of the contextual aspects of events, including spatial and temporal context [14], [24], [46], [47], as well as other types of source information [45], [48]–[52]. Finally, it has been reported that recognition performance in patients with schizophrenia relies to a far larger extend on familiarity than in healthy subjects [45], [53], [54].

Taken together, these studies show that patients with schizophrenia are impaired at using new links to retrieve an entire event from partial cues. However, this does not prove the wider claim made by Talamini et al. [15], [16], which states not only that binding disparate information is difficult for patients with schizophrenia, but also that their memory problems are *largely due* to binding deficits. It is the inability to form well-bound episodic representations that, according to Talamini et al. [15], [16], leads to deficits in recall. Published studies are mostly tangential to this issue, as they tend to compare recall of contextual information with recall of item information.

In contrast, the current study compares context conditions within one recall paradigm and allows us to investigate the effect of context processing on item recall itself. Surprisingly, we found no notable deficit in cued recall based solely on word stems, without the aid of context. As explained above, our model predicts this, because the word stem, as an intra-object cue, is relatively effective in a situation of decreased connectivity in the MTL. Our results suggest that, at least in samples of recent-onset patients, long-term memory deficits may be limited to the diminished effects of context.

Nevertheless, poor integration of event components in schizophrenia may have a profound influence on cognition. We have recently argued [16] that the effects are not limited to long-term memory. Instead, they may affect the way in which events are perceived in the first place, leading to problems in any task requiring the linking of stimuli over time and space. For instance, reduced MTL connectivity in our model produces a deficit in selecting subordinate responses over dominant ones based on context information. Deficits of this nature have been observed repeatedly in schizophrenia, for instance in lexical disambiguation [55]–[59] and ‘contextual’ versions of the Stroop task and continuous performance task [55], [60]. We have moreover argued that binding deficits may contribute to central schizophrenia symptoms such as contextually inappropriate behavior, associative abnormalities, conversational drift, concreteness and delusions [16].

In conclusion, we have demonstrated pronounced attenuation of context effects on retrieval in schizophrenia using a set-up that disentangles contextual memory processing from other aspects of memory. We found no difference in cued recall once contextual cueing was taken away. We thus conclude that contextual processing deficits may constitute a core dysfunction underlying the schizophrenia memory deficits profile. These findings corroborate the Talamini et al. [15], [16] model, in which reduced mediotemporal connectivity produces a binding deficit that is inextricably linked to a dominance of object information over spatial-configural aspects of events.
